# Differential effects of pine wilt disease on root endosphere, rhizosphere, and soil microbiome of Korean white pine

**DOI:** 10.1128/spectrum.02326-24

**Published:** 2025-03-06

**Authors:** Zehai Hou, Mingwei Wang, Hongwei Xu, Minggang Wang, S. Emilia Hannula

**Affiliations:** 1 The Key Laboratory for Silviculture and Conservation of Ministry of Education, College of Forestry, Beijing Forestry University, Beijing, China; 2 Key Laboratory of Beijing for the Control of Forest Pests, Beijing Forestry University 12380 , Beijing, China; 3 National Forestry and Grassland Administration Key Laboratory of Forest Resources Conservation and Ecological Safety on the Upper Reaches of the Yangtze River and Forestry Ecological Engineering in the Upper Reaches of the Yangtze River Key Laboratory of Sichuan Province, Sichuan Agricultural University, Chengdu, China; 4 Ecological Observation and Research Station of Heilongjiang Sanjiang Plain Wetlands, National Forestry and Grassland Administration, Shuangyashan, China; 5 Department of Environmental Biology, Institute of Environmental Sciences, Leiden University, Leiden, the Netherlands; University of the Philippines Los Baños, Laguna, Philippines; University of Houston-Downtown, Houston, Texas, USA

**Keywords:** pine wilt disease, *Pinus koraiensis*, fungal community, bacterial community, plant–soil compartment

## Abstract

Pine wilt disease (PWD), caused by pinewood nematodes, is highly destructive to pine forests in Asia and Europe, including Korean white pine (*Pinus koraiensis*). The microbiome in the needles and trunk of *Pinus* spp. are recognized to play key roles in resistance against PWD. However, the role of root and soil microbiomes in the resistance remains unclear. This study compares bacterial and fungal communities in the root endosphere, rhizosphere soil, and bulk soil of diseased versus healthy *P. koraiensis*. Results showed that PWD increased the α-diversity of fungi in rhizosphere soil but did not affect the microbial diversity in the root endosphere or bulk soil. The composition of bacterial and fungal communities in rhizosphere and bulk soils was significantly altered by PWD. Specifically, the relative abundance of Planctomycetes decreased, and the relative abundance of Tremellomycetes increased, while Agaricomycetes decreased in both rhizosphere and bulk soils after infestation with PWD, respectively. Relative abundances of Chloroflexi and Verrucomicrobia increased, while Proteobacteria decreased in bulk soil following PWD. Relative abundances of Leotiomycetes and Eurotiomycetes increased in the rhizosphere soil and bulk soil following PWD, respectively. Furthermore, with the host plant infestation by PWD, the relative abundance of ectomycorrhizal fungi decreases, while that of saprotrophic fungi increases in both rhizosphere and bulk soils. Our results revealed that PWD significantly affects the soil microbiomes of *P. koraiensis*, with varying impacts across different plant–soil compartments. This study provides insights into how root and soil microbiomes respond to PWD, enhancing our understanding of the disease’s ecological consequences.

**IMPORTANCE:**

The belowground microbiome is often sensitive to infection of forest diseases and is also recognized as a potential reservoir for selection of microbial agents against PWD. Our study demonstrates that the dynamics of belowground microbiome following natural infection of PWD are compartment and taxa specific, with varying degrees of responses in both diversity and composition of bacterial or fungal communities across the root endosphere, rhizosphere soil, and bulk soil. The results highlight the importance of utilizing appropriate plant–soil compartments and microbial taxa to understand the ecological consequences of the destructive PWD.

## INTRODUCTION

Above- and belowground organisms are separated in space but often intimately interact via their shared host plants (1). These aboveground–belowground biotic interactions occur due to systemic alterations in plant physiochemical traits incurred by the organisms (2). For instance, the removal of photosynthetic area by foliar herbivores often causes reduction in the amount of produced photosynthates and thus mitigates plant resource allocation to roots (3, 4). The mitigation may restrict the amount of food available to the belowground organisms and inhibit the synthesis and secretion of carbon-based metabolites by the roots (e.g., root exudates) that can feedback performance of the belowground organisms (5). Similarly, many belowground organisms, e.g., root-associated microbes, directly affect root growth and development by forming an antagonistic or symbiotic relationship or indirectly by modifying the water or nutrient cycling in the rhizosphere, resulting in changes in plant aboveground traits that determine colonization or performance of aboveground organisms (6, 7). The consequences of belowground organisms on their aboveground counterparts are well characterized (8), but studies on the other direction of these aboveground–belowground interactions are scarce (9, 10).

Plant-associated microbiomes, in particular the belowground microbiomes, have long been recognized as strong determinants of plant performance and fitness (11, 12). Compared to aboveground microbiomes, e.g., microbial communities residing in phyllosphere that are highly stochastic due to the open and fluctuating nature of the environment, belowground microbiomes are often more protected and can thus better reflect plant adaptation to the environmental changes (13). Belowground microbiomes are usually the sum of microbial communities that reside within plant roots (defined as “root endosphere”), in the root-soil interface (termed “rhizosphere”), and in soil that may not be directly influenced by plants (termed as “bulk soil”) (14, 15). Plant roots provide a foraging habitat for microbes in the endosphere (such as root endophytes) and affect the rhizosphere and soil microbes via exudation and rhizodeposition (16). Therefore, root and soil compartments typically harbor distinct assemblies of microbes, depending on their distance from roots and thus the magnitudes in influence by the roots (17–19). Many studies have shown changes in bacterial or fungal communities in a single plant or soil compartment following aboveground disturbances such as herbivory or pathogen infection (8, 20, 21). However, research on microbial communities across multiple compartments along the plant–soil interface in response to pathogen infection is largely missing (22, 23).

Pine wilt disease (PWD) is a devastating pine disease originating from North America. This disease has spread to large areas in Asia and Europe over the past decades, causing huge losses of pine forests in these regions (24, 25). PWD is caused by the pinewood nematode (PWN) *Bursaphelenchus xylophilus* (Steiner & Buhrer) vectored by long-horn beetles (*Monochamus* spp.) in nature (26). Once invaded in a pine tree, PWN can rapidly reproduce in the vascular system, causing the block of water transportation and killing the tree rapidly even in a few weeks (27). Several studies have shown that the assembly of microbial communities in the root endosphere or in the rhizosphere of different pine species is strongly altered by PWD, and in particular, fungal taxa seemed more affected than bacterial taxa by the occurrence or progression of this disease (28–30). For example, PWD caused a significant decrease in fungal richness but did not affect bacterial diversity in rhizosphere soil of Japanese black pine *Pinus thunbergia* (31). A similar result was shown in Masson pine, *Pinus massoniana*, that fungal diversity rather than bacterial diversity in rhizosphere soil was reduced by PWD infection, possibly due to the resultant decreases of ectomycorrhizal fungi but increases of saprotrophic fungi in their relative abundances (32). Yet, whether the microbial taxa altered by PWD are shared in different plant–soil compartments or to what extent this varies across the compartments remains unclear.

Korean white pine *Pinus koraiensis* Siebold & Zuccarini is an economically and ecologically valuable pine species and widely distributed in Northeast China (33). This species was recently found to be susceptible to PWD in the field (34). In this study, we compared microbial communities in the root endosphere, rhizosphere, and bulk soil from *P. koraiensis* that were either naturally infected by PWN or were uninfected to examine the patterns of belowground microbiomes across three plant–soil compartments of *P. koraiensis* in responses to natural occurrences of PWD. We hypothesize that (i) structures of the microbial communities across all the plant–soil compartments significantly differ between diseased and healthy *P. koraiensis* trees, and (ii) fungi were overall more affected by PWD than bacteria independent of the compartment identity because of their higher host specificity.

## MATERIALS AND METHODS

### Study site and pine species

In China, the original epidemic area of PWD was distributed in regions with mean annual temperatures above 10℃ (35). This temperature boundary was broken in recent years due to global warming, and accordingly, PWD tends to shift the distribution range toward North (36). Therefore, we conducted our study in Fushun, Liaoning province, Northeast China, in which natural occurrence of PWD was found recently (in 2017) and considered the northern border of the epidemic area of PWD in China (34). This study was performed in Dahuofang Forest Station in Fushun, which lies in a topographic basin at about 400–500 m above sea level. The experimental site is located in the middle of the temperate zone, where the climate is strongly influenced by China’s monsoons. In this region, the mean annual temperature is 5.5℃, and the mean annual precipitation is 661.22 mm, with an evaporation of 1,411.78 mm (37).

The Korean white pine *P. koraiensis* was used in our study. This species is a temperate evergreen tree species. It naturally adapts to moist monsoon climates with a relatively narrow ecological range. The natural distribution of *P. koraiensis* includes Southeast Siberia, Northeast China, Northern area of Korea, in addition to a few populations also found on the islands of Honshu and Shikoku in Japan (38, 39). The Korean white pine is graded as a second-class national preserved plant species in China (40). This pine species has been considered economically important for its values as sources of timber and pine oil (41). Nuts of the Korean white pine are also extensively used in medicine and the food market (42). Korean white pines in this region were recently found to be infected by PWN vectored by the Sakhalin pine sawyer *Monochamus saltuarius* Gebler (34). At the experimental site of the current study, the infection of Korean white pine by PWD typically begins in July, and a large proportion of the needles fall off after an average of 81 days after the infection (33).

### Experimental design

The *P. koraiensis* forests sampled in our study were established in the 1960s, and the majority of the forests were monoculture stands. One of these stands (41°57′44″N, 124°13′31″E) had been previously reported to be invaded by PWN vectored by *Monochamus saltuarius* (43, 44) and was selected as the “diseased” field site in our study. Another *P. koraiensis* stand that is located approximately 5.0 km away from the diseased site (41°58′38″ N, 124°16′41″ E) was regarded as the “healthy” site of *P. koraiensis* forests potentially as no symptoms of PWD had been found in this stand. Both experimental sites were hilly with slopes between 40° and 50° and an orientation from a south to north direction. The soil is of looseness and good perviousness with high capacity of retaining the fertility and water (45). The sampled soils contain 4.3–6.2 g/kg of soil organic carbon, 0.32–0.48 g/kg of total nitrogen, and 0.61–1.17 g/kg of total phosphorus. Within each stand, a sampling transect was made, along which at least 15 individuals of *P. koraiensis* pines with similar diameter at breast height (DBH) were randomly tagged. The tagged individuals were 15.5 ± 0.56 cm and 15.2 ± 0.55 cm in DBH for healthy and diseased trees, respectively (independent-sample *t*-test: *t*_1,28_ = 1.456, *P* = 0.157). Among the tagged trees, eight individuals with full green needles in the healthy site were selected, and eight individuals with a proportion of ca. 50% browning needles or slight wilting in the diseased site were selected (46). The distances between two selected individuals in each stand were at least 30 m. To verify the disease status of PWD, a piece of triangle-shaped tissue was taken from the trunk of each individual at breast height using a chain saw. The tissues were immediately transported to the laboratory and extracted using the “Baerman funnel method.” The microscopic examination showed that there were no *B. xylophilus* individuals in the tissues from tagged individuals in the healthy site, while the samples from individuals in the diseased site did contain *B. xylophilus* individuals. Six healthy and six diseased *P. koraiensis* trees were randomly selected from the confirmed individuals for soil sampling. The DBHs were 15.6 ± 0.48 cm and 15.4 ± 0.46 cm for the sampled healthy and diseased trees, respectively (independent-sample *t*-test: *t*_1,10_ = 0.676, *P* = 0.515).

### Sampling of pine roots and soils

For each of the selected individuals of *P. koraiensis*, the understory vegetation and litter were removed using a shovel that was previously sterilized using 75% alcohol. One soil core was taken from each direction at a distance of 0.5 m from the trunk using a soil sampler (diameter 38 mm) at a depth of 0–15 cm soil surface. The four soil cores from each individual tree were collected into a plastic bag and homogenized to be a composite sample, referred to as bulk soil. The first-class roots of the respective tree were also exposed using the shovel, and the connected lower-class roots were roughly shaken to remove a vast proportion of attached soil. The roots were detached from the higher-class roots using a sterilized scissor and further collected into a 15 mL plastic tube. The tubes were thoroughly shaken to separate the intimately attached soil from the roots, and the obtained soil in the tube was considered to be rhizosphere soil (47). The collected rhizosphere soil was transferred into a 2.0 mL Eppendorf tube, and the fine roots left in the 15 mL tube were used as the source of root endosphere. The used shovel and scissors under each individual tree were thoroughly sterilized at each sampling from these compartments (bulk soil, rhizosphere soil, and root endosphere). All the collected soil and root samples were stored in an ice box and immediately transported to the laboratory in Shenyang Institute of Technology, Shenyang, China.

In the laboratory, bulk soil samples were sieved through a surface-sterilized 2 mm sieve in diameter to remove large soil particles such as stones and roots and were transferred into 2.0 mL Eppendorf tubes. Both the bulk and rhizosphere soil samples were stored at –20°C for subsequent analysis. The collected root samples were washed under running water to thoroughly remove surface soils and placed in a centrifuge tube containing 30 mL phosphate buffer and sonicated in an ultrasonic cleaner (Jiemei-KS-5200DE, Kunshan, China) at 40 kHz for 1 min with five repetitions. The obtained root segments were further chemically decontaminated with 2% sodium hypochlorite for 5 min and washed five times using sterilized water to remove microbes residing on the rhizoplane. The treated root segments were further stored in liquid N and ground prior to DNA extraction.

### DNA extraction, PCR amplification, and sequencing

Total genomic DNA of root, rhizosphere soil, and bulk soil was extracted using the MoBio Power Soil DNA isolation kit (MoBio Laboratories, Carlsbad, CA, USA), according to the manufacturer’s protocol. The obtained DNA was analyzed by Majorbio Bio-Pharm Technology Co., Ltd. (Shanghai, China). Specifically, the final DNA concentration and purification were determined using a NanoDrop 2000 UV-vis spectrophotometer (Thermo Scientific, Wilmington, NC, USA) and DNA quality was examined via 1% agarose gel electrophoresis. The primers 338F (5′-ACTCCTACGGGAGGCAGCAG-3′)/806R (5′-GGACTACHVGGGTWTCTAAT-3′) were used to amplify V3–V4 regions of the bacterial 16S rRNA gene, and the primers ITS1-1 (5′-CTTGGTCATTTAGAGGAAGTAA-3′)/ITS1-2(5′-GCTGCGTTCTTCATCGATGC-3′) were used to amplify the fungal ITS1 region. All PCRs were performed using NEB Phusion High-Fidelity PCR Master Mix following the manufacturer’s recommendations with 30 ng of DNA, 4 µL of PCR primer cocktail, and 25 µL of PCR Master Mix. Negative controls (no templates) were included in this step to check for primer or sample DNA contamination. PCR products were verified by electrophoresis on a 1% agarose gel and purified using the Agencourt AMPure XP Beads Kit to remove unspecific products. The library quality was evaluated by an Agilent 2100 bioanalyzer instrument (Agilent DNA 1000 Reagents). The libraries were sequenced using an Illumina HiSeq platform (HiSeq SBS Kit V2, Illumina). Sequence data were deposited in the National Center for Biotechnology Information Sequence Read Archive database under accession number PRJNA1045839.

### Bioinformatics of sequencing data

The sequencing data were processed using Quantitative Insights Into Microbial Ecology (v.1.9.1), as described by Caporaso et al. (48). Raw fastq files were demultiplexed, quality-filtered by Trimmomatic, and merged by FLASH (v.1.2.7, http://ccb.jhu.edu/software/FLASH/) (49) according to the following standards: (i) reads were truncated at any site receiving an average quality score of <20 over a 50 bp sliding window; (ii) primers were matched, allowing two nucleotide mismatches, and reads containing ambiguous characters were discarded; and (iii) sequences with overlaps longer than 10 bp were merged based on their overlap sequence. Operational taxonomic units (OTUs) were clustered with a 97% similarity cutoff using UPARSE (v. 7.1, http://drive5.com/uparse/), proposed by Edgar (50). The chimeric sequences were identified and discarded using UCHIME (51). The taxonomy of each 16S rRNA and ITS sequence was analyzed using the Ribosomal Database Project Classifier algorithm, comparing against the Silva database (http://www.arb-silva.de/) released by Quast et al. (52) for 16S rRNA and the UNITE database (https://unite.ut.ee/) reported by Kõljalg et al. (53) for ITS, respectively.

Considering that the sequencing depth varied across samples, we further conducted a sub-sampling procedure to normalize the number of reads to the minimum observed within the same compartment between diseased and healthy *P. koraiensis* by the subSeq R package (54). The normalized number of reads for all samples is listed in .

### Functional analysis

The ecological functions of bacteria were predicted and annotated by the functional annotation of prokaryotic taxa approach using a rarefied OTU table (55). Most annotated species were assigned to groups involved in metabolic or ecological functions, such as C, N, and S cycling and the degradation of organic matter (55, 56).

To identify the variation in potential functions of the fungal communities across plant–soil compartments and PWD status, we used an open annotation tool FunGuild to assign the fungal taxa into three ecologically relevant trophic modes of saprotrophy, symbiotrophy, and pathotrophy. These modes were further divided into specific feeding guilds composed of fungi that share lifestyle characteristics, e.g., animal pathogens, endophytes, and mycorrhizae (57).

### Statistical analyses

α-Diversity was estimated for each sample by calculating two measures: observed features and Shannon index. The β-diversity of the microbial communities within each sample was calculated in order to estimate the differences among soil microbial community compositions, and the separation of microbial community composition between treatments was visualized using non-metric multidimensional scaling based on a Bray–Curtis dissimilarity matrix. Permutational multivariate analysis of variance (PERMANOVA) statistical tests were performed to determine the difference in community composition of bacteria and fungi in each plant–soil compartment between diseased and healthy pines using the “adonis” function in the “vegan” package, with 1999 permutations and using the Bray–Curtis distance matrix as input. The relative abundances of individual microbial taxa between healthy and diseased treatments within a compartment were compared by the Welch’s *t*-test in the Statistical Analysis of Metagenomic Profiles (v.2.1.3) software, and the differences were considered statistically significant at the *P* < 0.05 level (58). All the indices were calculated and analyzed using the vegan package, and the plots were made using the package “ggplot2” under R (v.4.0.1) (59).

## RESULTS

### Bacterial and fungal α-diversity

**Fig 1:**
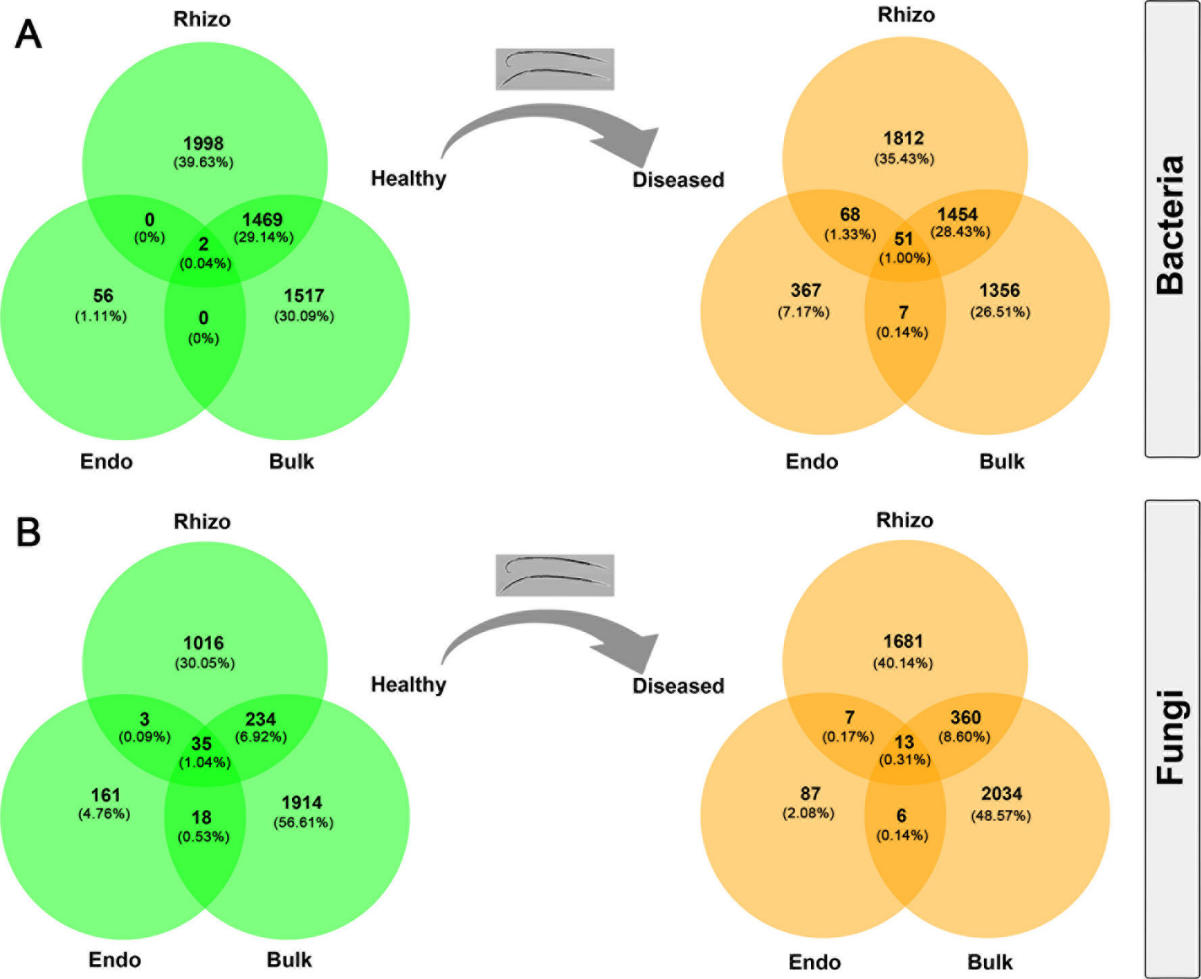
Venn diagrams showing the absolute numbers and percentage of unique and overlapped operational taxonomic units of (**A**) bacteria and (**B**) fungi among three plant–soil compartments (Endo, Rhizo, and Bulk) of healthy *P. koraiensis* and diseased *P. koraiensis* by pine wilt disease. Bulk, bulk soil; Endo, root endosphere; Rhizo, rhizosphere soil.

In total, there were 11,129 bacterial OTUs and 7,341 fungal OTUs detected from 36 samples across three plant–soil compartments of healthy and diseased trees. There were only two shared bacterial OTUs (0.04% out of the total number) among root endosphere, rhizosphere soil, and bulk soil from healthy pines, while 51 OTUs (1.00% out of the total number) were shared among the compartments from diseased pines (Fig. 1A). In contrast, the number of shared fungal OTUs in the root endosphere, rhizosphere soil, and bulk soil was higher in healthy pines (35 OTUs, 1.04%) than in diseased pines (13 OTUs, 0.31%, Fig. 1B). Both the number of unique bacterial and fungal OTUs in the root endosphere were substantially lower in healthy than in diseased pines, while the opposite was true for bulk soil and rhizosphere compartments (Fig. 1A and B).

**Fig 2:**
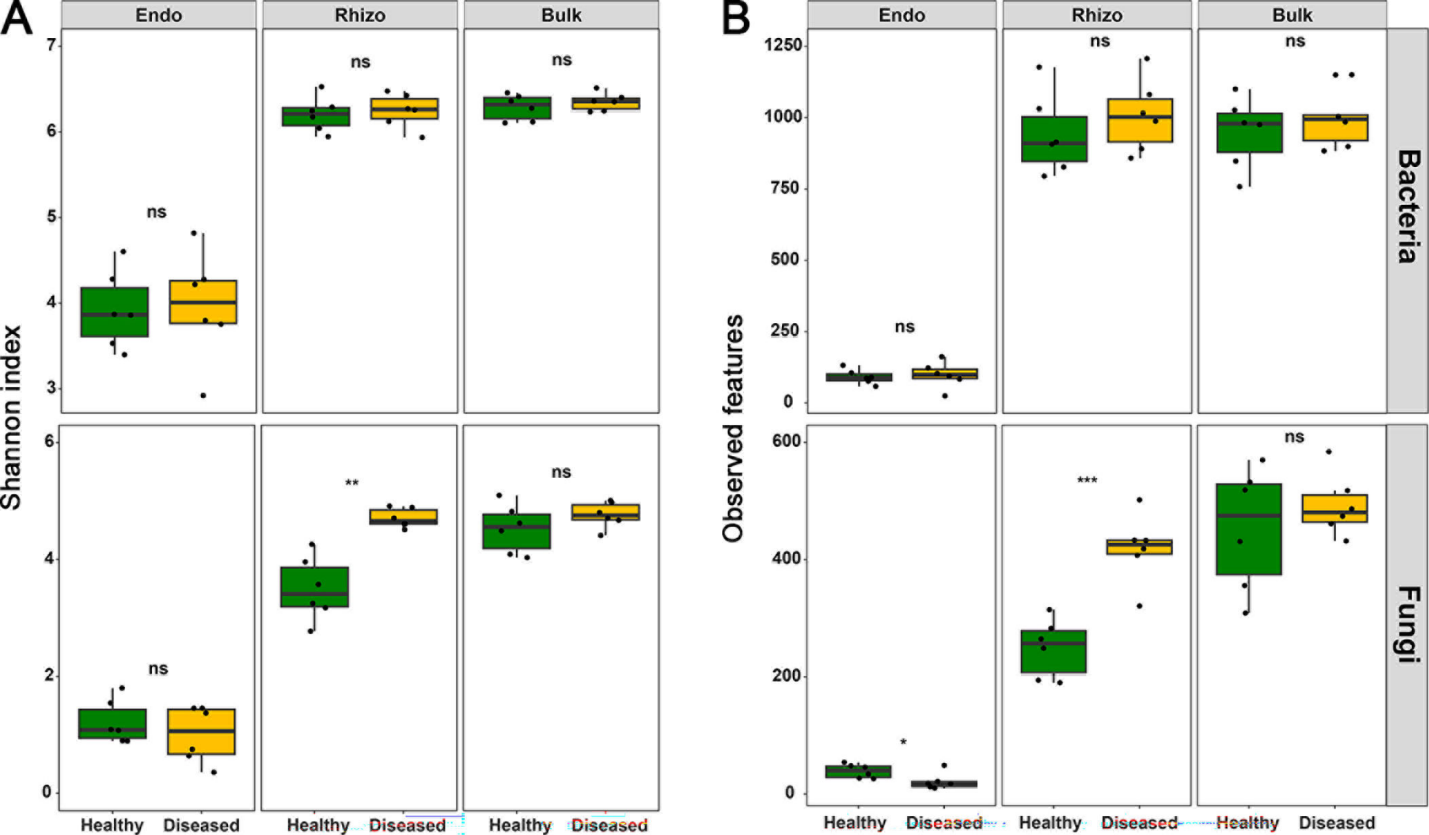
Box plots showing diversity indices for bacterial and fungal communities in three plant–soil compartments (Endo, Rhizo, and Bulk) of the healthy and diseased *P. koraiensis* by pine wilt disease. The diversity indices included (**A**) Shannon index and (**B**) observed features calculated from microbial operational taxonomic units (OTU) matrixes. The results are shown based on independent-sample *t*-tests. The number of asterisks indicated the level of significance: *P < 0.05, **P < 0.01, ***P < 0.001. Bulk, bulk soil; Endo, root endosphere; Rhizo, rhizosphere.

The α-diversity (measured by Shannon diversity and observed richness) of bacteria in root endosphere was significantly lower than that in rhizosphere soil and in bulk soil, but the latter two compartments did not significantly differ from each other. There were no differences in bacterial diversity between diseased and healthy pines in any of the compartments (all *P* > 0.05, Fig. 2). The α-diversity of fungi tended to increase with the increasing distance of examined compartment from the pine roots, and this was particularly true for healthy pines. The observed richness of fungi was lower in the root endosphere of the diseased as compared to healthy pines (*P* < 0.05, Fig. 2B), while the opposite trend was true for the rhizosphere soil in which both Shannon diversity and observed richness were significantly higher in diseased than in healthy pines (all *P* < 0.01, Fig. 2).

### Bacterial and fungal community compositions

**Fig 3:**
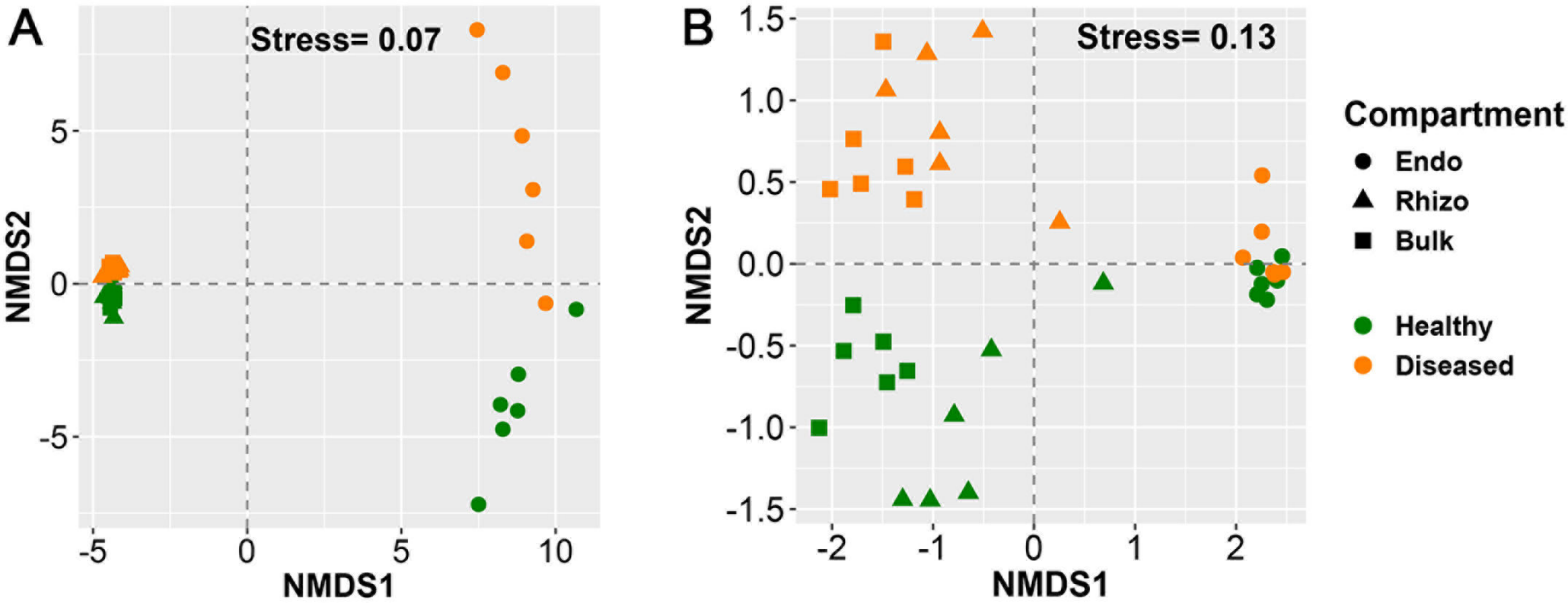
Ordination of non-metric multidimensional scaling (NMDS) based on Bray–Curtis dissimilarity for (**A**) bacterial and (**B**) fungal community composition in three plant–soil compartments of root endophere (Endo, circles), rhizosphere (Rhizo, triangles), and the bulk soil (Bulk, squares) from healthy (green) and diseased (brown) *P. koraiensis* by PWD. Stress values are included in the figure as a measure of “goodness of fit“ for the NMDS.

Both bacterial (PERMANOVAs: all *P* < 0.01, *R*^2^ = 0.36–0.37; Fig. 3A) and fungal (PERMANOVAs: all *P* < 0.01, *R*^2^ = 0.13–0.34; Fig. 3B) communities were significantly different in compositions between healthy and diseased pines across all the plant–soil compartments. For bacteria, the effects were equally strong across the compartments (*R*^2^ = 0.36–0.37), while for fungi, the effect was stronger in endopshere (*R*^2^ = 0.34) and bulk soil (*R*^2^ = 0.33) than in the rhizosphere (*R*^2^ = 0.13).

### Relative abundance of bacterial and fungal taxa

**Fig 4:**
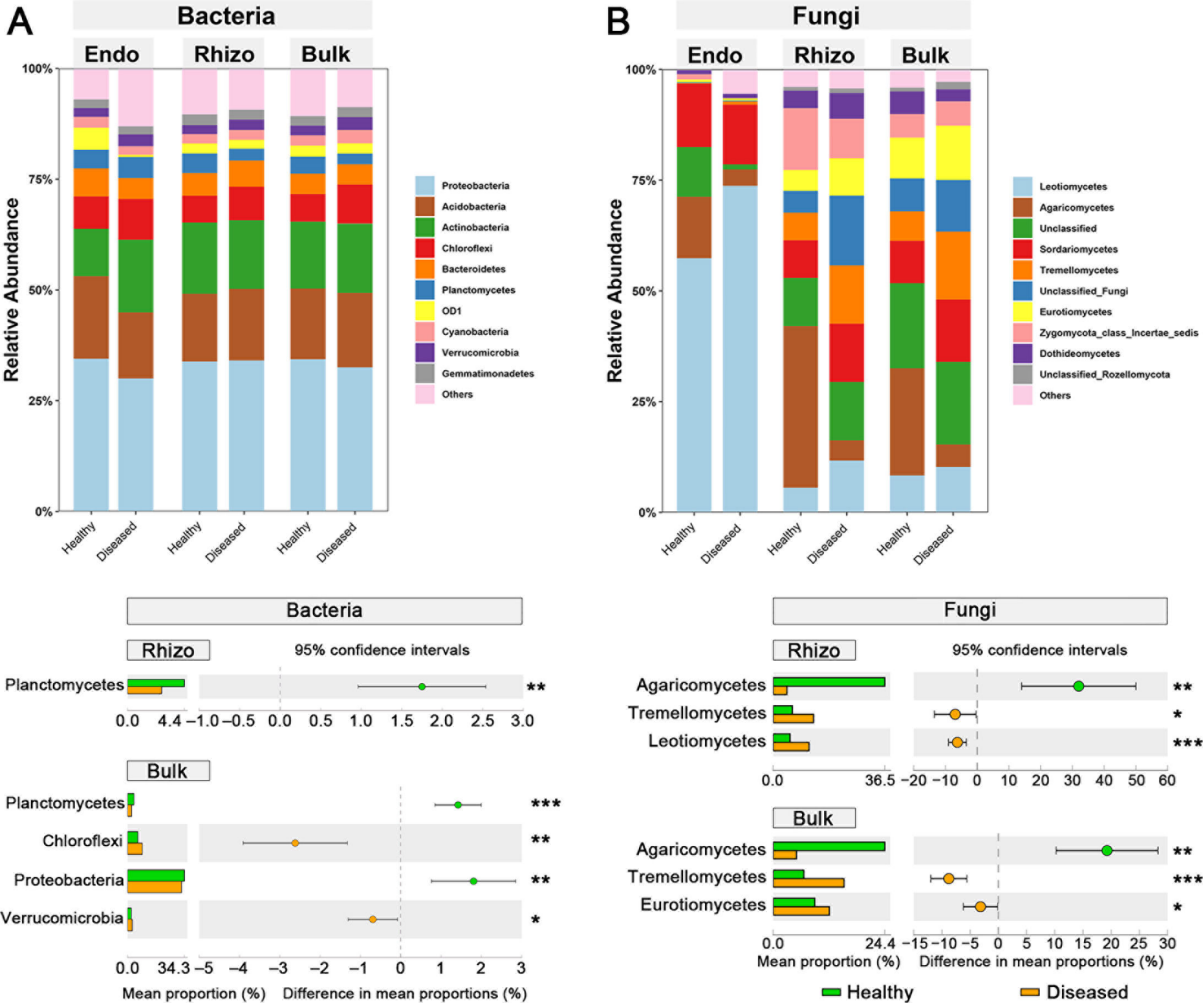
Relative abundances and the corresponding significant differences in dominant taxa that are composed of (**A**) bacterial communities at the phylum level, and (**B**) fungal communities at class level in three plant–soil compartments (Endo, Rhizo, Bulk) between healthy and diseased *P. koraiensis* by PWD. The most abundant 10 bacterial phyla and 10 fungal classes in microbial communities were averaged among replicated samples (*n* = 6). The bar plots represent the mean proportion of the phyla or class, and the colored circles and error bars represent the difference in mean proportion and the 95% confidence intervals, respectively. Significant difference in relative abundance of taxon between healthy and diseased *P. koraiensis* was assessed at *P* < 0.05 based on Welch’s *t*-test. The relative abundances of bacterial or fungal taxa in root endosphere (Endo) were not significantly different at the phylum or class level between healthy and diseased *P. koraiensis*, and thus are not shown in the figure. The number of asterisks indicates the level of significance. *P < 0.05, **P < 0.01, ***P < 0.001. Bulk, bulk soil; Rhizo, rhizosphere soil.

Bacterial communities in the root endosphere were dominated by phyla Proteobacteria, Acidobacteria, Actinobacteria, Chloroflexi, Bacteroidetes, and Planctomycetes that together represented ca. 80% of the total abundance in all compartments of both diseased and healthy trees (Fig. 4A). There was a lower relative abundance of Planctomycetes in rhizosphere soil from diseased pines than from healthy pines (Welch’s *t*-test: *P* < 0.01). Similarly, there were lower relative abundances of Planctomycetes and Proteobacteria in bulk soil from diseased than from healthy pines (Welch’s *t*-tests: both *P* < 0.01). On the contrary, the relative abundances of bacterial phyla Chloroflexi and Verrucomicrobia were higher in bulk soil from diseased pines compared to healthy pines (Welch’s *t*-tests: all *P* < 0.05, Fig. 4A). At the genus level, the relative abundances of *Burkholderia* and *Conexibacter* in rhizosphere and bulk soil, in addition to *Rhodoplanes* in bulk soil, were lower from diseased pines than from healthy pines (Welch’s *t*-tests: all *P* < 0.05, ).

The fungal community in the root endosphere was dominated by classes Leotiomycetes, Sordariomycetes, and Agaricomycetes, which together represented more than 75% of the total abundance. In rhizosphere soil and bulk soil, the fungal communities were dominated by the same classes, which accounted for 25%–50% of the total abundance (Fig. 4B). The relative abundance of Agaricomycetes was significantly higher in both rhizosphere and bulk soils sampled from healthy compared to diseased pines (Welch’s *t*-tests: all *P* < 0.01). In contrast, the relative abundance of Leotiomycetes and Tremellomycetes was significantly lower in rhizosphere soil of healthy than diseased pines (Welch’s *t*-tests: all *P* < 0.05). The relative abundance of Tremellomycetes and Eurotiomycetes abundance was significantly lower in bulk soil of healthy than diseased pines (Welch’s *t*-tests: all *P* < 0.05, Fig. 4B). There was higher relative abundance of *Cryptococcus* in rhizosphere and bulk soil from diseased than from healthy pines (Welch’s *t*-tests: *P* < 0.05). We did not observe significant differences in the relative abundance of fungal classes in root endosphere between healthy and diseased pines.

### Functional prediction analysis of bacteria and fungi

**Fig 5:**
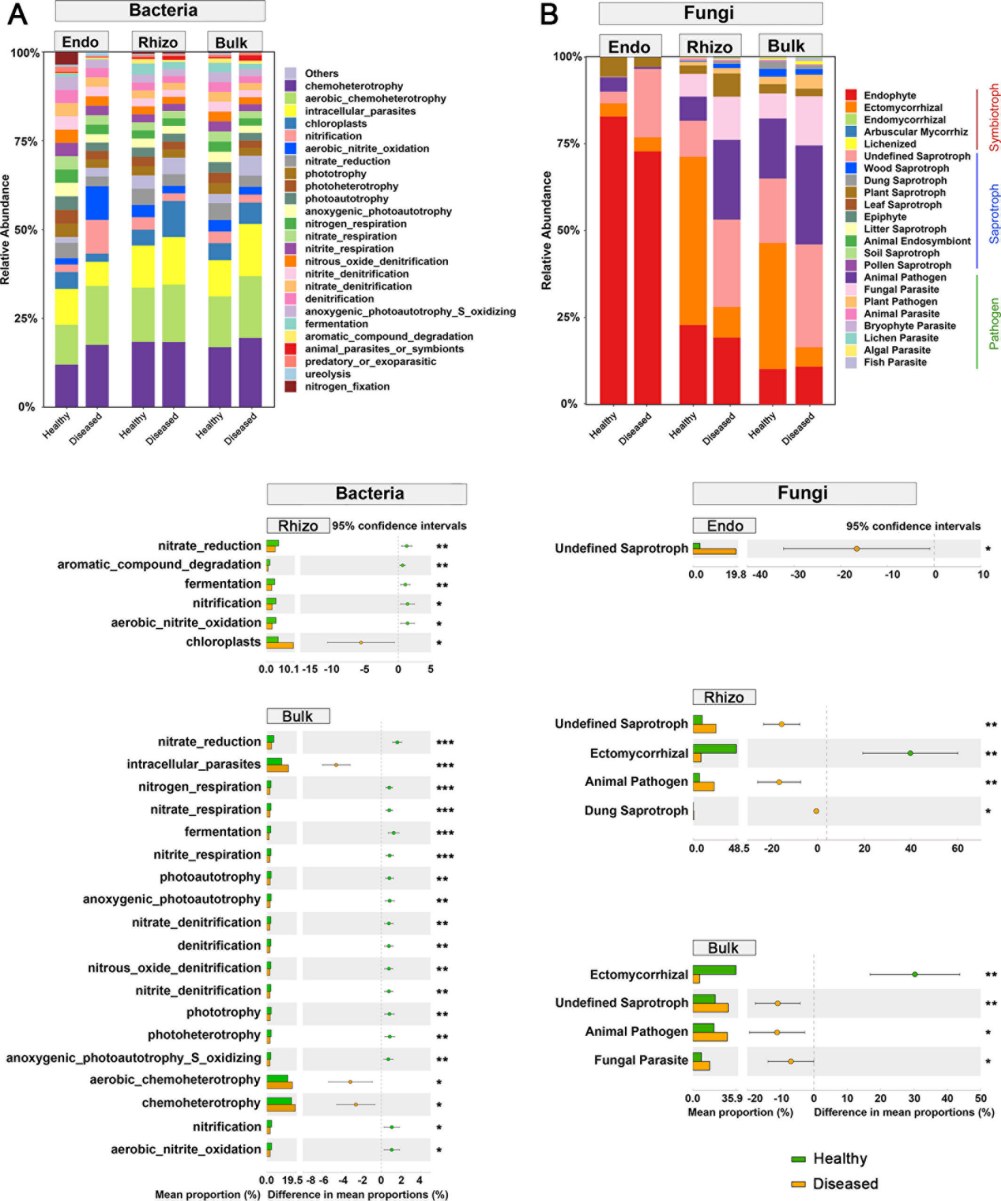
Relative abundances and the corresponding significant differences in (**A**) main soil bacterial ecological functions and (**B**) main soil fungal functional guilds in three plant–soil compartments (root endosphere, rhizosphere soil, and bulk soil) between healthy and diseased *P. koraiensis* by PWD. Low-abundance soil bacterial ecological functions with less than 0.5% of the total sequences across all samples are grouped into “others.” The bar plots represent the mean proportion, and the colored circles and error bars represent the difference in mean proportion and the 95% confidence intervals, respectively. Significant difference between treatments was assessed at *P* < 0.05 based on Welch’s *t*-test. The relative abundances of main soil bacterial ecological functions in root endosphere (Endo) were not significantly different between healthy and diseased *P. koraiensis* and thus are not shown in the figure. *P < 0.05, **P < 0.01, ***P < 0.001. Bulk, bulk soil; Endo, root endosphere soil; Rhizo, rhizosphere soil.

Bacterial OTUs were assigned to 64 functional groups, in which chemoheterotrophy and aerobic chemoheterotrophy were the most dominant functions across all samples (Fig. 5A). Although the relative abundance of OTUs related to the two functions did not differ in rhizosphere soil between diseased and healthy pines, it significantly increased in bulk soil of diseased pines compared to healthy ones (Fig. 5A). In addition, there were significant differences in relative abundances of OTUs in relation to other ecological functions, such as those related to N- and P-cycling and organic matter degradation in bulk soil between diseased and healthy pines (Fig. 5A).

Fungal OTUs were assigned to three ecologically relevant trophic modes, including saprotrophy, symbiotrophy, and pathotrophy, and each of them included multiple ecological guilds (Fig. 5B). In addition to undefined saprotrophs, other fungal guilds like animal pathogens or fungal parasites also had a higher relative abundance in diseased trees than in healthy trees across all three plant–soil compartments (Fig. 5B). We did not observe differences in relative abundance of Orbiliaceae, which was often considered nematophagous. The relative abundance of symbiotrophs and, in particular, ectomycorrhizal fungi was higher in rhizosphere and bulk soils under healthy trees as compared to soils under diseased trees (Fig. 5B).

## DISCUSSION

Foliar antagonists often systemically induce changes in physiological or chemical traits of belowground tissues of a plant (60). These changes may not only directly cause the succession of microbial community in root endosphere but also steer the communities in rhizosphere soil and in adjacent bulk soil via root exudation (13, 61, 62). Consequently, the microbial communities across multiple plant–soil compartments might be tremendously shifted by such foliar disturbances (63). In agreement with previous research, our study showed that the incidence of PWD caused by foliar nematode pathogens indeed strongly shifted the assemblies of both fungal and bacterial communities, although the direction and magnitudes of such shifts vary with the plant–soil compartments considered.

Earlier studies showed that the diversity of wood endophytic bacteria in the trunk of *Pinus pinaster* increased with the severity of PWD (64), yet the diversity of bacteria in rhizosphere and bulk soils decreased following PWD (32). Other studies showed that PWD may not necessarily affect the diversity of soil bacterial community under the host plant *P. thunbergii* (31, 65). Consistent with the latter findings, we did not find differences in bacterial α-diversity measured by both Shannon diversity and observed richness of species between the healthy and diseased pine trees in any of the three plant–soil compartments sampled. The contrasting results may be due to the differences in pine species, compartments, and ontogeny of the pines examined or the stages of PWD development among these studies (30, 64, 66). On the other hand, we found that the α-diversity of fungi in rhizosphere soil was consistently higher in diseased pines than in healthy pines, which is consistent with another study that also reported increased fungal α-diversity in the rhizosphere of *P. koraiensis* as a result of PWD (61). This increase in fungal diversity is mainly a result of enrichment of potentially necrotrophic fungi in the rhizosphere of diseased pines that were more recruited due to the decreased viability of diseased pines (67). However, it is noted that we did not find an overall difference in fungal α-diversity between diseased and healthy pines in root or in bulk soil. The result is in line with other studies in which the PWD did not distinguish the fungal community diversity in roots (31, 68), as endophytic fungi are often more conserved and less plastic than those in rhizosphere soil (17). The fungal community in the rhizosphere differed from that in the root endosphere or in bulk soil, which was not very surprising, given that the possible changes in root exudates as a result of PWD may more strongly affect fungal taxa in the rhizosphere than those in bulk soil (31). All these results advocate the complexity of three-way interactions among host pines, PWD, and microbes in the plant–soil interface, highlighting the necessity to further investigate the mechanisms underlying such interactions (31, 61, 69).

Interestingly, though there were no differences in diversity of bacterial communities, we noted that the composition of bacterial communities significantly differed between diseased and healthy *P. koraiensis* across all three plant–soil compartments. A similar result was reported in another study that investigated responses of the bacterial community in the rhizosphere of the same pine species to PWD (61). However, the composition of bacterial communities from other pine species, e.g., *P. massoniana* and *P. thunbergii*, seemed not affected by PWD (31, 65, 70). Obviously, consequences of bacterial communities following PWD may be highly host species specific or climate driven (30). On the other hand, in our study, fungal communities in rhizosphere and bulk soils were affected by PWD, while the community in root endosphere was not affected. Thus, fungal communities in soils of *P. koraiensis* appear more sensitive to PWD than those in root endosphere (31), which is in contrast to our hypothesis. This may be explained by root exudates being more responsive to PWD than root physiochemical traits, which further distinguish fungal communities of rhizosphere and bulk soils from the community in root endosphere of pines (71, 72). Therefore, future work should focus on the changes in root traits and root exudates or other attached soil properties induced by PWD to examine how these changes relate to the shifts in microbial communities at the plant–soil interface.

Relative abundances of bacterial phylum Planctomycetes in rhizosphere and bulk soils were decreased by PWD, which is consistent with the study of *P. massoniana* infected by PWD (73). Further analyses showed that within these altered phyla, genus *Conexibacter* was particularly lower in the soil under PWD. This change is in line with findings in another study, in which the relative abundance of *Conexibacter* was significantly lower in PWN-inoculated pine seedlings (74). *Conexibacter* has been found to enhance resistance of rhizosphere microregions to ecological toxicity and improve microbial community stability in stressed soils (75). The reduced abundance of *Conexibacter* by PWD may suggest a destructive soil function following this disease (71). Other bacteria such as *Rhodoplanes* or *Burkholderia* were more abundant in rhizosphere and bulk soils collected from healthy than from diseased pines (28, 74). These genera are often considered beneficial root symbionts to promote plant growth or protect plants against pathogen infections (76). The reduction in abundances of these microbes in the associated soils may also contribute to the wilting symptom or death of the diseased pines, in addition to the physiological dysfunction induced directly by the nematodes (74). Soil bacteria represented by chemoheterotrophy, aerobic chemoheterotrophy, or intracellular parasites were enriched, while those related to other functions, e.g., nutrient cycling, were reduced by the incidence of PWD in our study. Obviously, this disease diminishes the viability of the host pines and thus lessens the resistance against saprotrophs and nutrient requirement (77).

Leotiomycetes and Sordariomycetes from phylum Ascomycota, as well as Agaricomycetes from phylum Basidiomycota, were the most dominant fungal classes in all three plant–soil compartments, in line with findings of other studies that Ascomycota and Basidiomycota dominate the fungal communities in root endosphere, phyllosphere, and rhizosphere soil of *Pinus* spp. (30, 61, 78–80). We noted higher abundances of Agaricomycetes but lower abundances of Leotiomycetes and Tremellomycetes in the rhizosphere and bulk soils of healthy pines than the soils of diseased pines, suggesting a shift from slow-growing to fast-growing fungal species in the pine forest ecosystem following disturbance of PWD. The genus *Cryptococcus* within the Tremellomycetes class is able to infect and kill the nematode *Caenorhabditis elegans*, and the increase of its relative abundance indicates that the occurrence of PWD may enable the host pines to recruit beneficial fungi that serve biological control over pinewood nematodes (81). The relative abundance of fungal saprotrophs and potential animal pathogens or fungal parasites was elevated in soils by PWD. This may be because host pines are weakened by PWD, which often promotes the proliferation of saprotrophic organisms utilizing dead organic matter as food sources (82, 83). Conversely, we noted that relative abundances of ectomycorrhizal fungi were strongly reduced in soil from diseased pines, consistent with observations from infected Masson pine *P. massonian* by the disease (32). Such reduction may result from decoupling of the symbiosis between pine hosts and ectomycorrhizae as a result of PWD, considering that diseased pines may not be able to provide enough carbon to maintain the symbiotic relationships (69). The decreases in relative abundance of ectomycorrhizae following PWD would affect other flora and fauna in the vicinity by, e.g., altering the root exudates or reducing mycelial network complexity, which may indirectly contribute to the resistance of pines to PWD (84). Therefore, this result showed that PWD may promote its own occurrence and development by altering the outcome of interactions between host pines and soil microbes.

### Conclusions

To our knowledge, this study is among the first studies aiming to compare assemblies of belowground microbial communities among multiple plant–soil compartments following incidence of PWD. The findings highlight that the profile and potential functions of the belowground microbial communities in *P. koraiensis* forest can be strongly altered by the incidence of PWD, but the patterns of such alterations not only differ between bacterial and fungal communities, but also among the compartments from which these microbial communities were collected. To clarify the factors underlying such taxa- or compartment-specific patterns of belowground microbial communities observed in this study, future work should include investigation of shifts in root metabolomes, rhizodeposition, as well as soil nutrients under the pine forests following the incidence of PWD. Collectively, our research advanced the understanding of patterns in belowground microbial responses to PWD, and the results may provide insights into assessing the ecosystem-level consequences of this disease.

## Data Availability

The sequencing data generated in this study have been deposited in National Center for Biotechnology Information’s Sequence Read Archive (SRA) database (https://submit.ncbi.nlm.nih.gov/subs/sra) and are accessible through SRA series accession number PRJNA1045839.
